# Route of Inoculation Determines Symptom Profile and Replication Dynamics After low Pathogenic Avian Influenza A(H7N9) Virus Infection in Ferrets

**DOI:** 10.1111/irv.70183

**Published:** 2025-11-02

**Authors:** Sook‐San Wong, Mark Zanin, Min‐Suk Song, Cristina Contreras, Thomas P. Fabrizio, Eun‐Kyo Hong, Hye Kwon Kim, Woonsung Na, Richard J. Webby, Sun‐Woo Yoon

**Affiliations:** ^1^ HKU‐Pasteur Research Pole LKS Faculty of Medicine, the University of Hong Kong Hong Kong SAR China; ^2^ School of Public Health LKS Faculty of Medicine, the University of Hong Kong Hong Kong; ^3^ Centre for Immunology & Infection Hong Kong; ^4^ Chungbuk National University College of Medicine Cheongju Republic of Korea; ^5^ Department of Host‐Pathogen Interactions St. Jude Children's Research Hospital Memphis Tennessee USA; ^6^ Department of Vaccine Biotechnology Gyeongkuk National University Andong Republic of Korea; ^7^ Department of Microbiology Chungbuk National University Cheongju Republic of Korea; ^8^ School of Dentistry, Seoul National University Seoul Republic of Korea

**Keywords:** ferret, H7N9, influenza virus, pathogenicity, route of inoculation

## Abstract

**Background:**

Although influenza A viruses (IAVs) are respiratory pathogens, infections may occur via nonrespiratory routes. However, the effects of different routes of exposure on the course of infection and disease are not well characterized.

**Methods:**

This study assessed the pathogenicity and host responses in ferrets inoculated with the low pathogenic A/Anhui/1/2013 (H7N9) influenza A virus via different routes. Ferrets were inoculated through various routes, and viral replication in the respiratory tract was evaluated using nasal wash samples as well as respiratory and nonrespiratory tissues. Host immune responses were analyzed using peripheral blood collected from the virus‐inoculated ferrets.

**Results:**

Inoculation of ferrets with H7N9 via the intranasal (IN), intraocular (IO), or intraesophageal (IE) routes revealed that IN inoculation led to the greatest distribution of virus, whereas IO and IE inoculation led to more restricted viral spread. However, despite different routes, the respiratory tract remained the preferred site of IAV replication. IN‐ and IE‐inoculation led to greater symptom severity compared to IO inoculation. Proinflammatory cytokine expression was highest in IN‐inoculated ferrets and was not always associated with cumulative viral loads.

**Conclusions:**

Overall, these results indicated that the route of inoculation can influence tissue distribution and disease outcomes, but the respiratory tract remains the primary site of viral replication.

## 1 Introduction

In mammals, influenza A viruses (IAVs) primarily infect via the respiratory route. However, infections via other routes, such as the eye or the gastrointestinal tract, have also been observed and documented experimentally and can lead to symptomatic influenza. Cases of ocular infections have been observed following the exposure to subtype H7 IAVs, and more recently, following exposure to unpasteurized milk from cows infected with the recent Clade 2.3.4.4b A(H5N1) viruses, a highly pathogenic avian influenza A virus (HPAIV) [1, 2]. Ingestion of contaminated animal products can also increase the risk of IAV infection. Fatal systemic infections have been reported in domestic cats fed unpasteurized colostrum and milk from H5N1 infected cows [3]. Furthermore, poultry skeletal muscle can harbor H5N1 IAVs in asymptomatic animals and ingestion of chicken meat contaminated with subtype H5N1 HPAIV can lead to infection in ferrets, although higher viral doses are required compared to respiratory transmission [4, 5, 6, 7]. Infection of dogs and cats with IAV following the consumption of infected poultry has also been documented [8, 9]. Therefore, while IAV infection of mammals via different routes of inoculation (ROIs) has been documented, it is not known how different ROI affect the course of infection, disease, or host responses in mammals. To address this, we inoculated ferrets with a low pathogenic subtype H7N9 IAV via the intranasal (IN), intraesophageal (IE), and intraocular (IO) routes and compared the pathogenicity, virus dissemination, tissue‐specific viral adaptations and host responses elicited by each ROI.

## 2 Methods and Materials

### 2.1 Virus Propagation, Titration, and Cell Culture

The low pathogenic influenza virus A/Anhui/1/2013 (H7N9) was provided by the Chinese Center for Disease Control and Prevention. Virus stocks were propagated in 9‐ to 10‐day‐old specific pathogen free embryonated chicken eggs and titrated by egg infectious dose 50% (EID_50_). Viral titers in the ferret samples were determined by tissue culture infectious dose 50% (TCID_50_) in Madin‐Darby canine kidney (MDCK) cells (ATCC) [10]. MDCK cells were cultured in minimal essential medium (MEM) supplemented with 10% fetal calf serum (FCS), vitamins, 2 mM L‐glutamine, and antibiotics (Thermo Fisher Scientific) in a humidified 5% CO_2_ environment at 37°C. For the titration of MDCK cells, viruses were diluted in the infection medium (MEM supplemented with 5% bovine serum albumin [Sigma]). The limit of detection was 2 log_10_ TCID_50_/mL. Statistical differences were determined by two‐way analysis of variance (ANOVA) using GraphPad Prism (GraphPad Software Inc.).

### 2.2 Ferret Experiments

Three‐ to five‐month‐old male ferrets (Triple F Farms, Sayre, Pennsylvania, USA) were housed in accordance with the Institutional Animal Care and Use Committee guidelines. The experiments were performed in biosafety level 3 (BSL‐3) containment. All ferrets were tested for prior influenza virus infection by screening for anti‐influenza virus antibodies via hemagglutination inhibition against the influenza virus strains A/Tennessee/1‐560/2009 (H1N1), A/Perth/16/2009 (H3N2), and A/Anhui/1/2013 (H7N9). Prior to inoculation, baseline body temperature and weight were recorded. The animals were anesthetized with isoflurane and inoculated with 10^6^ EID_50_ of A/Anhui/1/2013 (H7N9) in 1 mL of PBS with antibiotics (Sigma) for inoculation via the IN or IE route or in 100 μL of PBS with antibiotics for inoculation via the IO route. A total of eight ferrets were used for each inoculation route. IN inoculations were performed by administering 0.5 mL of inoculum dropwise into each nostril via a pipette. IE inoculations were performed by gavage using an 18‐gauge, 3‐in. rounded bulb gavage needle. IO inoculations were performed by administering 50 μL of inoculum dropwise onto the surface of each eye via a pipette.

Nasal washes were collected from each ferret on 1, 3, 5, 7, and 11 days post inoculation (DPI) by flushing the nostrils with 1 mL of PBS. Clinical signs, including weight loss, fever, and nasal discharge, were monitored every alternate day for 11 DPI. To study tissue tropism, two ferrets inoculated via the IN, IO, and IE routes were euthanized according to institutional protocols on 3, 5, 7, and 11 DPI. Respiratory tract tissues (nasal turbinate, trachea, and lungs) and nonrespiratory tract tissues (stomach, liver, mediastinal lymph nodes, spleen, kidney, small intestine, olfactory bulb, and conjunctiva) were collected and the tissues weighed and then homogenized using a TissueLyser II (QIAGEN). Homogenates were clarified by centrifugation and titers were determined using TCID_50_. Blood samples were collected for immunological profiling as described in Section 2.3. Cumulative viral loads (CVL) were calculated by summing the viral titers detected in all tissue samples from individual ferrets to estimate the total viral burden in each.

### 2.3 Measurement of Cytokine Profiles

RNA was extracted from 2.5 mL of whole blood using PAXgene Blood RNA Tube (QIAGEN). The washed cell pellet was resuspended in 1 mL TRIzol (Invitrogen) and RNA purified using the Direct‐zol RNA MiniPrep Kit (Zymo Research) according to the manufacturer's instructions. Reverse transcription was performed using 1 μg RNA, SuperScript III reverse transcriptase (Invitrogen), and random primers (Invitrogen) according to the manufacturer's instructions. Quantitative PCR was performed using the SYBR Green PCR Master Mix (QIAGEN) and a 7500 Fast Quantitative PCR System (Applied Biosystems). The housekeeping gene β‐actin was used as an endogenous control. The following cytokines were analyzed: interleukin (IL)‐2, −4, −6, −8, and −10, tumor necrosis factor α (TNF‐α), interferon (IFN) α, β, and γ using gene‐specific primers (Table 1) [11, 12]. Differences in expression greater than two‐fold in mean relative quantitation (RQ) compared to that in samples collected from mock‐infected ferrets after normalization to *GAPDH* expression were considered statistically significant.

**TABLE 1:** Sequences of primers used to detect ferret cytokine/chemokine gene expression.

Gene	Primer sequence (5′‐3′)
*IL‐2*	Fwd: TGCTGCTGGACTTACAGTTGCTCT Rev.: CAATTCTGTGGCCTTCTTGGGCAT
*IL‐4*	Fwd: CGTTGAACATCCTCACAGCGAGAAAC Rev.: TTGCCATGTTCCTGAGGTTCCTGTGA
*IL‐6*	Fwd: CAAATGTGAAGACAGCAAGGAGGCA Rev.: TCTGAAACTCCTGAAGACCGGTAGTG
*IL‐8*	Fwd: AACCCACTCCACGCCTTTCCATC Rev.: GGCACACCTCTTTTCCATTGAC
*IL‐10*	Fwd: TCCTTGCTGGAGGACTTTAAGGGT Rev.: TCCACCGCCTTGCTCTTATTCTCA
*TNF‐α*	Fwd: ATGCTCCTGCGACAAATGAGGAGA Rev.: TTCTGCAGCTGCTTGCTGTCAAAC
*IFN‐α*	Fwd: ATGCTCCTGCGACAAATGAGGAGA Rev.: TTCTGCAGCTGCTTGCTGTCAAAC
*IFN‐β*	Fwd: GGTGTATCCTCCAAACTGCTCTCC Rev.: CACTCCACACTGCTGCTGCTTAG
*IFN‐γ*	Fwd: CCATCAAGGAAGACATGCTTGTCAGG Rev.: CTGGACCTGCAGATCATTCACAGGAA
*GAPDH*	Fwd: TTGCTGACAATCTTGAGGGAGTT Rev.: CTGCTGATGCCCCCATGT
*Beta‐actin*	Fwd: GCAGGTCATCACCATCG Rev.: TGGAGTTGAAGGTGGTCT

### 2.4 Statistical Analyses

Differences between groups were assessed using analysis of variance (ANOVA) or Student *t* tests using GraphPad Prism v8. *P* values < 0.05 were considered statistically significant.

## 3 Results

### 3.1 Subtype H7N9 IAVs Inoculated via Different Routes Replicated in Respiratory Tract Tissues and Caused Influenza Symptoms

**FIGURE 1:**
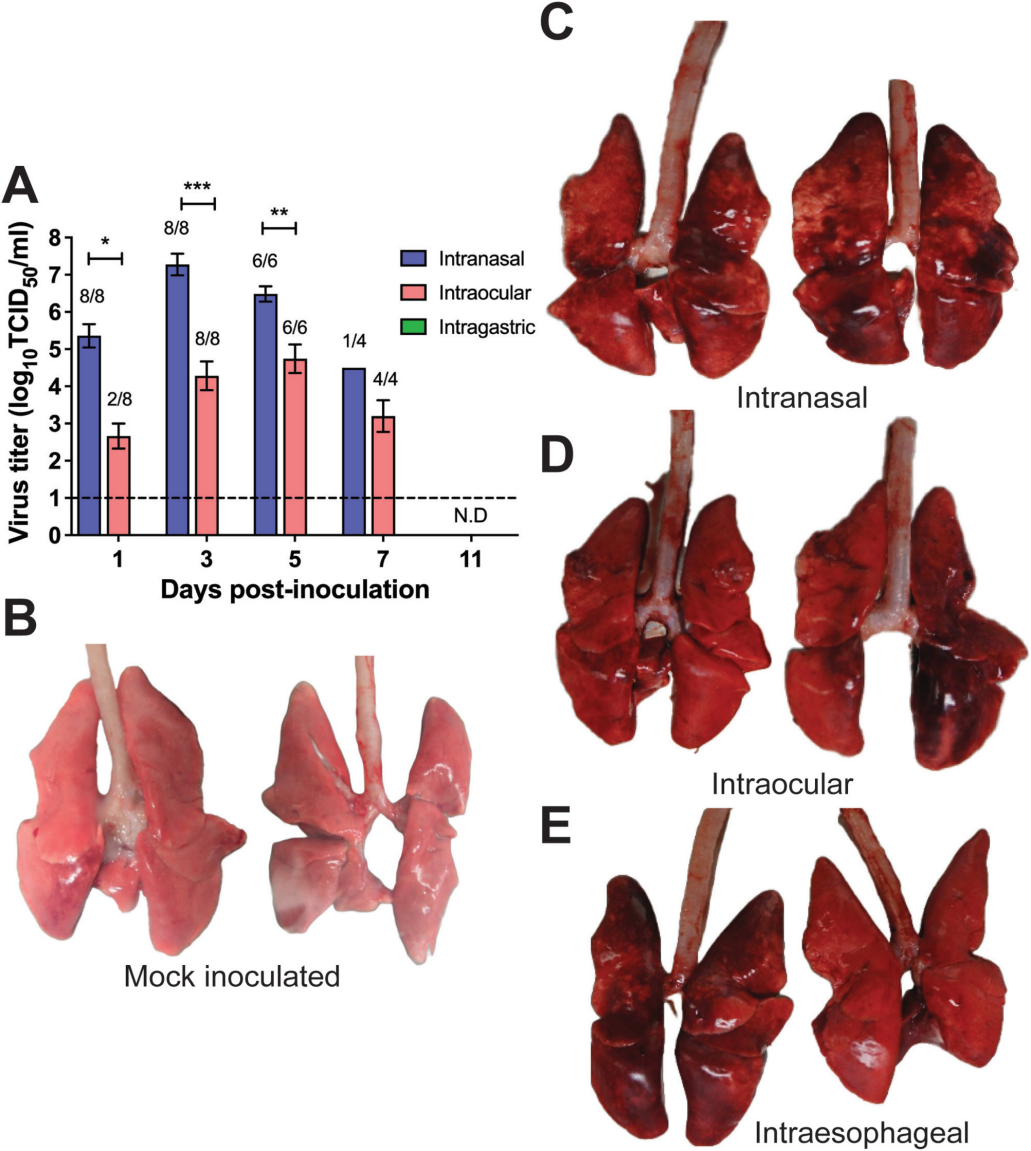
A/Anhui/1/2013 (H7N9) was detected in nasal washes obtained from ferrets inoculated intranasally and intraocularly, but not intraesophageally, yet lung pathology was observed in ferrets inoculated via all these routes. Viral titers in nasal washes were detected more consistently and at higher titers in intranasally inoculated ferrets than in intraocularly inoculated ferrets, and titers were not detected in any nasal washes obtained from intraesophageally inoculated ferrets (A). Compared to lungs from mock inoculated ferrets (B), gross lung pathology consistent with influenza virus infection was observed in the lungs obtained from intranasally (C), intraocularly (D), and intraesophageally (E) inoculated ferrets at seven days post inoculation (*n* = 2 ferrets). N.D., not detected. *p < 0.05. **p < 0.01. ***p < 0.001.

Virus was detected in the nasal washes of all IN‐inoculated ferrets at 1, 3, and 5 DPI, in one of the four ferrets at 7 DPI, and in no ferrets at 11 DPI. Virus was detected in the nasal washes collected from two IO‐inoculated ferrets at 1 DPI, and in those collected from all IO‐inoculated ferrets at 3, 5, and 7 DPI, but not at 11 DPI. In contrast, viruses were not detected in any nasal washes collected from IE‐inoculated ferrets at any time point, although clinical signs of influenza were observed (Figure 1A).

Weight loss in IN‐ and IE‐inoculated ferrets was similar, at 3.75 ± 1.34% and 2.17 ± 0.71% starting body weight, respectively. IO‐inoculated ferrets lost less weight, at 0.45 ± 1.29%, although this difference was not statistically significant (Table 2). Increased body temperatures were observed in all IN‐inoculated ferrets, and in five IO‐ and five IE‐inoculated ferrets, although these differences were not statistically significant. Respiratory symptoms were observed in six IN‐inoculated ferrets compared to two IO‐ and four IE‐inoculated ferrets. Neurological symptoms were absent in all ferrets (Table 2). Overall, influenza symptoms were observed in IN‐, IO‐, and IE‐inoculated ferrets, being more frequent in IN‐inoculated ferrets.

**TABLE 2:** Clinical signs observed in ferrets inoculated with A/Anhui/1/2013 (H7N9).

Route of inoculation	No. ferrets showing indicated clinical symptom/total number			
	Weight loss^a^	Temperature increase^a^	Respiratory symptoms^b^	Neurological symptoms^c^
Intranasal	7/8 (3.75 ± 1.34)	8/8 (3.04 ± 0.42)	6/8	0/8
Intraocular	2/8 (0.45 ± 1.29)	5/8 (1.72 ± 0.57)	2/8	0/8
Intraesophageal	7/8 (2.17 ± 0.71)	5/8 (2.75 ± 1.02)	4/8	0/8

^a^ Maximal changes in the mean value (percentage) ± SD are shown in parentheses.

^b^ Respiratory symptoms were sneezing, labored breathing, and nasal discharge. Criteria for inclusion were the observation of at least one symptom during the course of the experiment.

^c^ Neurological symptoms were hind limb paresis, ataxia, torticollis, and tremors. Criteria for inclusion were the observation of at least one symptom during the course of the experiment.

Next, we studied viral replication and tissue tropism in respiratory tract tissues and nonrespiratory tract tissues. We calculated the cumulative viral loads (CVLs) of all tissues samples to gain an indication of total viral burdens. At 3DPI the highest CVLs were evident in IN‐inoculated ferrets, followed by IO‐ and IE‐inoculated ferrets, at 28.6 ± 2.1, 19.7 ± 3.4 and 13.4 ± 2.4 TCID_50_/g, respectively (Table 3). Gross pathology of ferret lungs at 7 DPI was consistent with IAV infection, including edema and pulmonary consolidation accompanied by mucopurulent exudates (Figure 1B–E). Viral titers were detected in the lungs of IN‐inoculated ferrets at 3 and 5 DPI but not at 7 DPI, whereas IO inoculation did not appear to robustly result in lung infection, with comparatively low titers detected in one ferret at 3 and 5 DPI only. Interestingly, viral titers were detected in the trachea and lung of IE‐inoculated ferrets and lung viral titers in IE‐inoculated ferrets were higher than those in IN‐inoculated ferrets at 3 DPI but were not detected at later time points (Table 4). These data therefore indicated viral spread from the gastrointestinal tract to the respiratory tract. Viral replication was detected in the trachea, with IN‐inoculation associated with relatively high viral titers at 3 and 5 DPI but not at 7 DPI. Viral titers were only detected in one IO‐inoculated ferret at 5 DPI and were also detected in IE‐inoculated ferrets at 3 and 5 DPI at values comparable to those measured in IN‐inoculated ferrets.

**TABLE 3:** Cumulative viral loads of all tissues sampled in individual ferrets inoculated with A/Anhui/1/2013 (H7N9).

Days post inoculation	Intranasal inoculation^a^		Intraocular inoculation		Intraesophageal inoculation	
3	26.5	30.7	16.3	23.2	15.8	11
5	30	N.D.^b^	25.7	17.2	4.2	N.D.
7	N.D.	2.7	11.9	N.D.	9.1	N.D.

^a^ Cumulative viral loads (TCID_50_/g of tissue) of individual ferrets were calculated by summing the viral titers detected in all tissue samples to estimate total viral burdens.

^b^ N.D. not determined.

**TABLE 4:** Titers of A/Anhui/1/2013 (H7N9) detected in ferret tissues.

Route of inoculation	Days post inoculation	Animal ID	Virus titer (log_10_ TCID_50_/g tissue)^a^											
			Nasal turbinate	Trachea	Lung	Stomach	Liver	Lymph node	Spleen	Kidney	Intestine	Esophagus	Brain	Conjunctiva
Intranasal	3	F250	7.1	4.4	3.3	—	—	—	—	—	—	3.8	5.1	2.7
		F251	7.6	5.7	4.4	—	—	—	—	—	—	4.6	5.7	2.7
	5	F261	7.4	6.1	6.0	—	—	—	—	—	—	4.4	6.2	—
		F262	4.9	5.1	4.2	—	—	—	—	—	—	4.9	7.3	—
	7	F263	—	—	—	—	—	—	—	—	—	—	—	—
		F264	2.7	—	—	—	—	—	—	—	—	—	—	—
Intraocular	3	F257	6.9	—	3.2	—	—	2.1	—	—	—	4.2	—	—
		F258	6.2	—	—	—	—	—	—	—	—	5.3	6.2	5.5
	5	F265	7.6	—	3.9	—	—	—	—	—	—	4.0	6.1	4.0
		F266	8.2	3.1	—	—	—	—	—	—	—	3.3	2.7	—
	7	F248	6.8	—	—	—	—	—	—	—	—	—	5.1	—
		F249	7.1	—	3.9	2.2	—	—	—	—	—	—	5.4	—
Intraesophageal	3	F267	—	5.5	6.0	—	—	—	—	—	—	4.3	—	—
		F268	—	5.2	5.8	—	—	—	—	—	—	—	—	—
	5	F253	—	4.2	—	—	—	—	—	—	—	—	—	—
		F254	—	—	—	—	—	—	—	—	—	—	—	—
	7	F246	4.9	—	—	—	—	—	—	—	—	4.2	—	—
		F247	—	—	—	—	—	—	—	—	—	—	—	—

^a^ Dashes indicate values at or below the limit of detection (2 log_10_TCID_50_/g).

Relatively high viral titers of 5.15 ± 1.25 TCID_50_/g and 6.9 ± 0.3 TCID_50_/g were detected at 3 DPI in the nasal turbinates of IN‐ and IO‐inoculated ferrets, respectively. However, at 7 DPI, relatively low titers were detected in only one IN‐inoculated ferret. Relatively high titers were detected in the nasal turbinates of both IO‐inoculated ferrets at 7 DPI (Table 4). In contrast, viral titers were only detected in one IE‐inoculated ferret at 7 DPI, suggesting that viral replication at this site was delayed. Relatively high viral titers were detected in the esophagus at 3 and 5 DPI, but not at 7 DPI, in IN‐ and IO‐inoculated ferrets and in one IE‐inoculated ferret at 3 and 7 DPI (Table 4).

Viral titers were detected in the olfactory bulb of IN‐inoculated ferrets at 3 and 5 DPI, but not at 7 DPI. In contrast, in IO‐inoculated ferrets, viral titers were detected at 3 DPI (in one out of two ferrets), 5 DPI and 7 DPI. Viral titers were not detected in the olfactory bulb or conjunctiva of IE‐inoculated ferrets at any time point. Viral titers in the conjunctiva were detected in two IN‐inoculated ferrets at 3 DPI and one IO‐inoculated ferret at both 3 and 5 DPI (Table 4).

Viral titers were not detected in the liver, spleen, kidney, or small intestine of any ferret, including IE‐inoculated ferrets (Table 4). While virus was detected in the stomach of one ferret and the lymph nodes of another, both IO‐inoculated, they were close to the limit of detection. Because of the small sample sizes, statistical analyses of these viral titer data were limited. Overall, these data indicated that, despite different ROIs, the respiratory tract remained the main site of efficient IAV replication. Compared to IE inoculation, the IO route resulted in viral dissemination to the upper respiratory tract and brain but neurological symptoms were not observed.

### 3.2 Systemic Inflammation Showed Different Temporal Kinetics That Did Not Necessarily Track With Total Viral Burden

**FIGURE 2:**
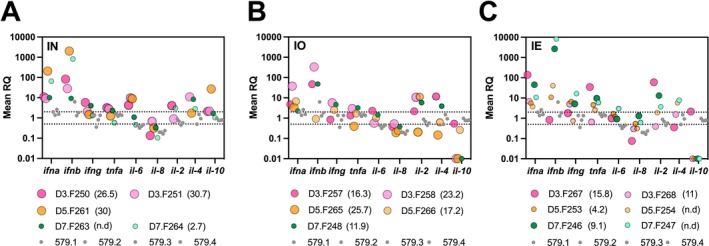
Expression of cytokine/chemokine genes in the peripheral blood of ferrets inoculated with A/Anhui/1/2013 (H7N9) via different routes in relation to cumulative viral loads. *IFN‐α* and *IFN‐β* gene expression was the most upregulated in ferrets inoculated by intranasal (A), intraocular (B), or intraesophageal (C) routes at days three, five, and seven post inoculation (D3, D5, and D7, respectively), although the kinetics of the response profile differed across the different routes of inoculation. Individual ferrets are colored according to the legend, on days post inoculation when samples were collected. The size of the symbol indicates the cumulative viral loads detected (indicated in parentheses) in all tissues at sampling time. *N* = 2 ferrets per time point. Samples from a mock‐infected ferret (579) were included as internal controls with data from each run (*n* = 4) as indicated.

To study host responses in A/Anhui/1/2013 (H7N9)‐inoculated ferrets, we measured the expression profiles of a panel of nine cytokine and chemokine genes in peripheral blood using samples from mock‐infected ferrets as the baseline. Of the 10 genes studied, the expression of IFN‐α and IFN‐β were most consistently upregulated in infected ferrets, albeit with varying kinetics and intensity depending on the ROI (Figure 2). Pro‐inflammatory genes were upregulated between 10‐ and more than 1000‐fold at 3 and 5 DPI in IN‐ and IO‐inoculated ferrets (Figure 2). Notably, the magnitude of gene expression did not necessarily track with CVL at the time of sampling, particularly in IE‐inoculated ferrets. For example, CVLs were generally lower in IE‐inoculated ferrets, as reflected by the size of the symbols in Figure 2, and in Table 3. However, apart from the delayed onset, gene expression profiles were similar to those in IN and IO ferrets.

IFN‐β expression was upregulated between 1000‐ and 10,000‐fold in IE‐ferrets at 7 DPI. Other genes that showed differential expression profiles were IL‐6 and TNF‐α. IL‐6 expression was 4‐ to 10‐fold higher in IN‐inoculated ferrets at 3 and 5 DPI but did not increase appreciably beyond 2‐fold in any IO‐ or IE‐inoculated ferrets. In contrast, TNF‐α expression showed a 2.5‐ to 34‐fold increase in five of six IE‐inoculated ferrets compared to IN‐ or IO‐inoculated ferrets. IL‐10 gene expression was downregulated in IO‐ and IE‐, but not in IN‐inoculated ferrets, whereas the expression of the chemokine IL‐8 was consistently undetected in most inoculated ferrets. Taken together, greater increases in the expression of pro‐inflammatory cytokine genes were observed in samples taken at earlier timepoints from IN‐ and IO‐inoculated ferrets compared to IE‐inoculated ferrets. While greater TNF‐α expression was detected in IE‐inoculated ferrets, it was overall more variable and did not seem to be associated with CVLs.

## 4 Discussion

Subtype H7 IAVs pose a continuous threat to health, as evidenced by numerous reports of outbreaks in animals and humans. Compared to other avian IAV subtypes, such as H5N1 and H9N2, a greater diversity of H7 avian IAVs subtypes, both of low and high pathogenicity, have caused infections and outbreaks in humans. Human cases of low pathogenic H7N2, H7N3, H7N4, H7N7, and H7N9, and highly pathogenic H7N3, H7N7, and H7N9 have already been reported. In poultry, outbreaks caused by H7N2, H7N3, H7N4, H7N7, H7N8, and H7N9 have been reported in several countries across continents, with significant impacts on the poultry industry [13, 14].

The propensity of infection via extra‐respiratory sites in humans, particularly the IO route, is associated more with subtype H7 IAVs than with other subtypes [15]. In mice, extra‐respiratory spread of the low‐ and highly pathogenic subtype H7 avian IAVs had been reported previously [16]. Furthermore, IO inoculation of subtype H7N9 IAVs in ferrets from the first three waves of the H7N9 outbreak in China led to respiratory tract infections [17]. IO‐inoculation of IAVs in ferrets, including A/Shanghai/1/2013 (H7N9) isolated during the first H7N9 wave in China, can lead to infection of the respiratory tract and the olfactory bulb and then to onward transmission via direct contact and respiratory droplet spread, albeit at lower frequency compared to IN inoculation [18]. Unlike infection via the IO route, the IE route has not been associated with subtype H7 IAVs. However, the consumption of meat contaminated with IAV can result in infection anyways. Experimentally, IE inoculation of highly pathogenic H5N1 avian influenza viruses in cats can lead to systemic spread, likely facilitated by viral tropism in endothelial cells and possibly the multibasic cleavage site of these IAVs [19, 20]. Fatal systemic infections have also been reported in domestic cats fed unpasteurized colostrum and milk from cows infected with clade 2.3.4.4 H5N1, suggesting similar systemic spread from the gastrointestinal tract [3]. However, the IE inoculation of guinea pigs with highly pathogenic H5N1 avian IAVs did not lead to systemic spread [21]. While these ROI have been studied, to the best of our knowledge, no study has yet compared how subtype H7N9 IAVs replicate and spread from different inoculation sites and how the disease course may be affected. Therefore, we compared the infectivity of A/Anhui/1/2013 (H7N9) in ferrets following inoculation via the IN, IO, and IE routes.

IN inoculation was associated with higher viral titers in nasal washes and tissues of the respiratory tract than in extra‐respiratory ROI. However, respiratory tract infections following extra‐respiratory inoculation were also evident. Nasal‐wash viral titers were also observed following IO inoculation, as shown in previous studies, but with lower mean titers [18]. Viral titers in nasal washes from only two of the eight IO‐inoculated ferrets were detected at 3 DPI, however, at later time points, the virus was detected in nasal washes from all IO‐inoculated ferrets. Viral titers were detected in the nasal washes collected from all IN‐inoculated ferrets at all‐time points. However, they were not detected in the nasal washes or nasal turbinates at any time point following IE inoculation. A delay in the detection of viral titers in nasal washes may be expected, considering that the inoculation site is farther from the respiratory tract compared to IO inoculation. However, relatively high viral titers were detected in the lungs and trachea at 3 DPI via the IE route, the earliest time point studied, and in the trachea of one ferret at 5 DPI, but not at 7 DPI. Therefore, a delay in viral replication in the respiratory tract following IE inoculation compared to that after IN or IO inoculation was not apparent. These data indicated the presence of a host factor between the upper and lower respiratory tracts that prevented detectable viral replication in the upper respiratory tract. This host factor may be the temperature differential of 33°C and 37°C between the upper and lower respiratory tracts, respectively, that can limit the transmissibility of avian influenza viruses adapted to the warmer temperatures of the avian gastrointestinal tract [22]. However, further studies may reveal host‐virus interactions that restrict viral infection or replication. Overall, although both IN and IO inoculations could lead to onward transmission via respiratory secretions, it seems unlikely that this could occur following IE inoculation, despite the evidence of replication in the respiratory tract. This would need to be confirmed through ferret transmission studies.

Interestingly, no virus was detected in the intestine following IE inoculation. IAVs can replicate in human intestinal epithelial cells, and gastrointestinal infections have been observed in humans [23, 24, 25]. Furthermore, relatively few viruses have been detected in the gastrointestinal tract of ferrets IN‐inoculated with A/Anhui/1/2013 (H7N9) or A/Shanghai/1/2013 (H7N9) [26]. It is possible that infection of the intestine may require a longer timeframe or that anatomic barriers, such as the stomach, limit the spread of IAVs following inoculation via the oral/esophageal routes. It was also interesting that virus titers were not detected in any nasal wash samples collected from IE‐inoculated ferrets yet virus titers could be detected in the trachea and lung at 3 and 5 DPI and even in the nasal turbinate at 7 DPI in IE‐inoculated ferrets (Figure 1A and Table 4). Pathology studies of these tissues may provide insights into viral replication in these tissues in the apparent absence of shedding.

Although our cytokine expression dataset was limited by small sample size, a trend of delayed inflammatory responses following IE inoculation was evident. IE‐inoculated ferrets showed high expression of TNF‐α, IFN‐β, and IFN‐γ at 7 DPI, which seemed to be related to the decline in IN and IO ferrets. Increased TNF‐α levels have been observed in pediatric cases of rotavirus‐induced diarrhea [27]. Paneth cells, which are secretory epithelial cells in the intestine, have been shown to produce TNF‐α and may be important contributors to the host response to IAV following IE inoculation [28]. Furthermore, proinflammatory responses have been observed in human intestinal epithelial cells infected with H9N2 avian IAVs. Interestingly, this study also showed a stronger inflammatory response to these avian IAVs than to the 2009 pandemic subtype H1N1 IAV [23]. In this study, we showed that IE inoculation led to systemic responses. Further studies would be required to characterize the mechanisms underlying this response.

## 5 Conclusions

Our study demonstrated that exposure to IAVs at ocular or intragastric sites carries the risk of respiratory infection that could potentially lead to onward transmission. Our study revealed that the respiratory tract remained the dominant site of IAV replication even after extra‐respiratory inoculation. The mechanisms underlying how extra‐respiratory ROIs lead to respiratory tract infection, particularly following IE inoculation, have the potential to reveal the virus‐host interactions underlying IAV infection and may provide insights into the propensity of IAVs to infect via extra‐respiratory routes.

## Author Contributions

**Sook‐San Wong:** conceptualization, investigation, funding acquisition, writing – original draft, formal analysis. **Mark Zanin:** conceptualization, investigation, funding acquisition, writing – original draft. **Min‐Suk Song:** conceptualization, formal analysis. **Cristina Contreras:** investigation. **Eun‐Kyo Hong:** data curation. **Hye Kwon Kim:** investigation. **Woonsung Na:** data curation. **Richard J. Webby:** conceptualization, supervision, funding acquisition, writing – review and editing. **Sun‐Woo Yoon:** conceptualization, investigation, funding acquisition, writing – original draft, writing – review and editing.

## Ethics Statement

All animal studies were approved by the Animal Care and Use Committee of St. Jude Children's Research Hospital (protocol 464).

## Conflicts of Interest

The authors declare no conflicts of interest.

## Peer Review

The peer review history for this article is available at https://www.webofscience.com/api/gateway/wos/peer‐review/10.1111/irv.70183.

## Data Availability

All data generated or analyzed during this study are deposited in figshare, URL: https://figshare.com/articles/figure/Figures_resubmission/30164755.
